# A randomized study to evaluate the safety and immunogenicity of a pentavalent meningococcal vaccine

**DOI:** 10.1038/s41541-024-00935-8

**Published:** 2024-08-07

**Authors:** Yoonjin Kim, Sungyeun Bae, Kyung-Sang Yu, SeungHwan Lee, Chankyu Lee, Jinil Kim, Howard Her, Jaeseong Oh

**Affiliations:** 1 Department of Clinical Pharmacology and Therapeutics, Seoul National University College of Medicine and Hospital, Seoul, Republic of Korea; 2 R&D Division, EuBiologics Co., Ltd, Seoul, Republic of Korea; 3 Department of Pharmacology, Jeju National University College of Medicine, Jeju, Republic of Korea; 4 Clinical Research Institute, Jeju National University Hospital, Jeju, Republic of Korea

**Keywords:** Drug development, Meningitis

## Abstract

A randomized, active-controlled, double-blind, first-in-human, phase 1 study was conducted in healthy Korean adults to evaluate the safety, tolerability, and immunogenicity of EuNmCV-5, a new pentavalent meningococcal vaccine targeting serogroups A, C, W, X, and Y. Sixty participants randomly received a single dose of either EuNmCV-5 or MenACWY-CRM, a quadrivalent vaccine containing serogroups A, C, W, and Y. Safety was assessed through monitoring anaphylactic reactions, adverse events for 28 days, and serious adverse events over 180 days. Immunogenicity was assessed via rabbit complement-dependent serum bactericidal antibody (rSBA) assay. EuNmCV-5 was safe, well-tolerated, and elicited a substantial antibody titer increase. The seroprotection rates exceeded 96.7%, and the seroconversion rates were over 85% for all the targeted serogroups. It showed higher seroconversion rates against serogroups A and C (*p* = 0.0016 and 0.0237, respectively) and elicited a substantial increase in GMT for all targeted serogroups compared to the MenACWY-CRM.

ClinicalTrials.gov identifier: NCT05739292.

## Introduction

*N. meningitidis* is a gram-negative bacterium, usually residing harmlessly in the human upper respiratory tract as a commensal^1^. In a small proportion of carriers, the pathogen can enter the bloodstream, causing septicemia or meningitis. Invasive meningococcal disease can rapidly progress to death within 48 h of symptom onset^2^. Additionally, 10–20% of survivors of meningococcal meningitis develop long-term neurologic sequelae such as hearing loss, intellectual disability, or other neurological disorders^3^. While invasive meningococcal disease frequently appears in small outbreaks, specific regions often experience devastating epidemics. This is the case in the African meningitis belt, the region in sub-Saharan Africa stretching from Senegal in the west to Ethiopia in the east^4^.

*N. meningitidis* is classified into 12 serogroups based on the antigenic differences of their capsular polysaccharides^1^, and six serogroups (A, B, C, W, X, Y) cause the most invasive cases globally^5^. In the African meningitis belt, serogroup A was the primary cause of the disease. However, since the implementation of meningococcal vaccines targeting serogroup A in 2010, no confirmed cases of serogroup A have been reported in the meningitis belt since 2017^6^. Recent meningococcal outbreaks in the region have mostly been due to serogroups C and W, with some reports also indicating outbreaks caused by serogroup X^6,7^.

Currently, most of the licensed meningococcal vaccines do not provide protection against serogroup X^8,9^, and Men5CV (MenFive^®^, Serum Institute of India Pvt. Ltd., Pune, India) is the only vaccine targeting serogroup X. It held pre-qualification from the World Health Organization (WHO) in July 2023. WHO’s Strategic Advisory Group of Experts on Immunization recommended the inclusion of Men5CV in the routine immunization programs of all countries within the meningitis belt^10^. While Men5CV can be an efficient solution, it requires reconstitution before administration with a provided diluent. This process may be perceived as cumbersome, suggesting a need for further developments in vaccine technology to create more readily deployable alternatives. Given this, EuNmCV-5, developed by EuBiologics Co., Ltd. (Seoul, Republic of Korea), is a pentavalent (A, C, W, X, and Y) meningococcal conjugate vaccine that includes coverage for serogroup X, using a non-toxic mutant of diphtheria toxin (cross-reactive material 197, CRM_197_) as a carrier protein. It is designed as a single vial, eliminating the reconstitution step. This clinical study aimed to evaluate the safety, tolerability, and immunogenicity of this new pentavalent meningococcal vaccine in comparison with an active comparator in healthy Korean adults who had not previously been vaccinated against or exposed to *N. meningitidis*.

## Result

### Participants

**Fig. 1:**
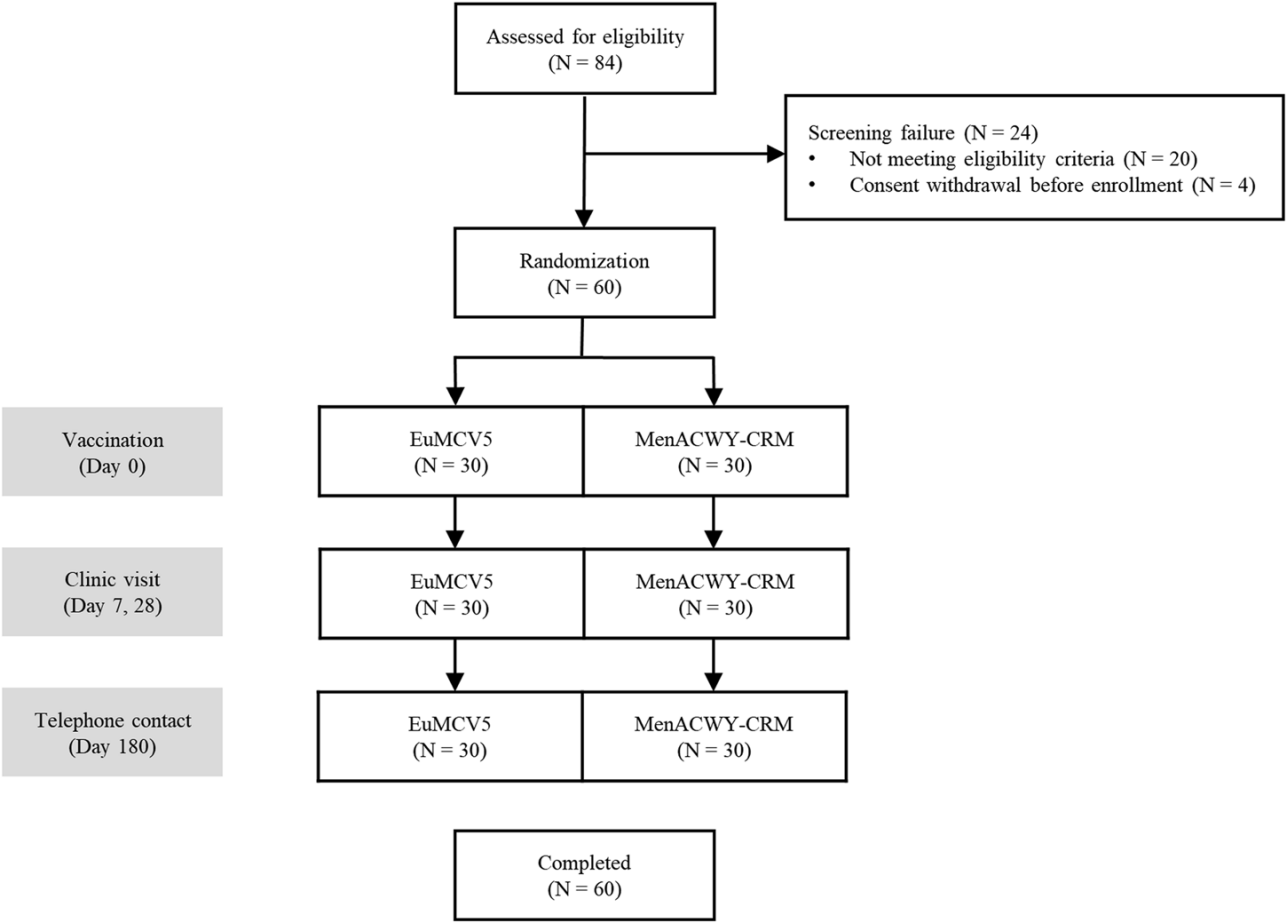
Study flow and disposition. Sixty participants were randomly assigned in a 1:1 ratio to either the intervention vaccine, EuNmCV-5, or the control vaccine, MenACWY-CRM. Clinic visits were scheduled 7 and 28 days after vaccination. A follow-up telephone contact was made 180 days after vaccination.

Sixty participants were randomized and completed the study as planned (Fig. 1). The first participant was screened on March 9, 2023, and the last participant finished the study on October 4, 2023. Most of the participants (76.7%) were females. The mean age at baseline was 39.9 and 39.2 years in the EuNmCV-5 and MenACWY-CRM groups, respectively. Sex, age, and body weight were well distributed between both groups (Table 1).

**Table 1:** Demographics of the safety analysis set

	EuNmCV-5 (*N* = 30)	MenACWY-CRM (*N* = 30)
Sex		
Female, *N* (%)	24 (80.0)	22 (73.3)
Male, *N* (%)	6 (20.0)	8 (26.7)
Age (years)	39.9 ± 8.2	39.2 ± 10.2
Race		
Asian, *N* (%)	30 (100.0)	30 (100.0)
Body weight (kg)	67.1 ± 14.5	60.2 ± 9.5

Data are presented as the number of participants (%) or mean ± standard deviation.

### Safety and tolerability

No anaphylactic reaction was reported within 30 min after vaccination, and none of the participants experienced any life-threatening adverse events. All adverse events were self-limited and resolved without sequelae. The incidence and severity of adverse events were comparable between the EuNmCV-5 and MenACWY-CRM groups. Each adverse event occurred only once per participant.

The most frequently reported solicited local injection-site reaction within 7 days of vaccination was tenderness, occurring in 11 participants (36.7%) in the EuNmCV-5 group and 6 participants (20.0%) in the MenACWY-CRM group (Table 2). Among the 30 cases of solicited local injection-site reactions, 18 cases were reported in the EuNmCV-5 group and 12 cases in the MenACWY-CRM group. Within the EuNmCV-5 group, one case was graded as moderate in tenderness, and another was as severe urticaria. All solicited local injection-site reactions in the MenACWY-CRM group were mild in severity. The most frequently reported solicited systemic reactions were fatigue/malaise and myalgia (Table 2). In the EuNmCV-5 group, both fatigue/malaise and myalgia were reported by 5 participants (16.7%), and all cases were mild in severity. In the MenACWY-CRM group, 4 participants (13.3%) experienced fatigue/malaise, and an equal number reported myalgia. One case in each category was graded as moderate. Within 28 days of vaccination, the most frequently reported unsolicited adverse event was rhinorrhea, observed in 4 participants (13.3%) in both the EuNmCV-5 and MenACWY-CRM groups (Table 2). There were no clinically significant changes in clinical laboratory tests and vital signs before and after the administration of the study vaccines.

**Table 2:** Solicited and unsolicited adverse events within 28 days of vaccination

	EuNmCV-5 (N = 30)	MenACWY-CRM (N = 30)
Solicited adverse events (Local injection site reactions)		
Pain	6 (20.0)	3 (10.0)
Mild	6 (20.0)	3 (10.0)
Moderate	0	0
Tenderness	11 (36.7)	6 (20.0)
Mild	10 (33.3)	6 (20.0)
Moderate	1 (3.3)	0
Erythema/redness	0	1 (3.3)
Mild	0	1 (3.3)
Moderate	0	0
Induration/swelling	0	0
Mild	0	0
Moderate	0	0
Urticaria	1 (3.3)	2 (6.7)
Mild	0	2 (6.7)
Moderate	0	0
Severe	1 (3.3)	0
Solicited adverse events (Systemic reactions)		
Fever	0	0
Mild	0	0
Moderate	0	0
Headache	2 (6.7)	4 (13.3)
Mild	1 (3.3)	3 (10.0)
Moderate	1 (3.3)	1 (3.3)
Fatigue/malaise	5 (16.7)	4 (13.3)
Mild	5 (16.7)	3 (10.0)
Moderate	0	1 (3.3)
Nausea	3 (10.0)	1 (3.3)
Mild	3 (10.0)	1 (3.3)
Moderate	0	0
Vomiting	0	0
Mild	0	0
Moderate	0	0
Myalgia	5 (16.7)	4 (13.3)
Mild	5 (16.7)	3 (10.0)
Moderate	0	1 (3.3)
Arthralgia	0	0
Mild	0	0
Moderate	0	0
Chills	0	1 (3.3)
Mild	0	0
Moderate	0	1 (3.3)
Rash	1 (3.3)	0
Mild	1 (3.3)	0
Moderate	0	0
Acute allergic reaction	0	0
Mild	0	0
Moderate	0	0
Unsolicited adverse events^a^		
Rhinorrhea	4 (13.3)	4 (13.3)
Mild	4 (13.3)	4 (13.3)
Moderate	0	0
Sneezing	2 (6.7)	0
Mild	2 (6.7)	0
Moderate	0	0
Lymphadenopathy	1 (3.3)	2 (6.7)
Mild	1 (3.3)	2 (6.7)
Moderate	0	0
Headache	2 (6.7)	0
Mild	2 (6.7)	0
Moderate	0	0

Data are displayed as the number of participants (percentage of participants).

^a^Unsolicited adverse events that occurred in more than 5% of participants were presented.

### Immunogenicity

Although people with a history of meningococcal vaccination, previous meningococcal infection, or recent contact with a person with meningococcal infection within the last 2 weeks were excluded, some participants had high baseline titers before vaccination. This was particularly true for serogroup A (16 out of 30 participants (53.3%) in the EuNmCV-5 group, 18 out of 30 participants (60.0%) in the MenACWY-CRM group).

Twenty-eight days after vaccination, seroconversion against all serogroups was observed in the majority of participants in the EuNmCV-5 group. Specifically, for serogroup X, 26 out of 30 participants (86.7%) in the EuNmCV-5 group demonstrated seroconversion, while only 2 out of 30 participants (6.7%) in the MenACWY-CRM group showed seroconversion (*p* < 0.001). The EuNmCV-5 group exhibited a higher seroconversion rate against serogroups A and C compared to the MenACWY-CRM group (*p* = 0.0016 and 0.0237, respectively) and it showed similarly high seroconversion rates (greater than 90%) in serogroups W and Y (Table 3).

**Table 3:** Immunogenicity against each serogroup assessed by rSBA

	EuNmCV-5 (*N* = 30)		MenACWY-CRM (*N* = 30)		*p* value
	*N*	% (95% CI)	*N*	% (95% CI)	
Seroconversion^a^					
A	27	90.0 (79.3–100.0)	16	53.3 (35.5–71.2)	0.0016
C	30	100.0 (100.0–100.0)	24	80.0 (65.7–94.3)	0.0237
W	30	100.0 (100.0–100.0)	27	90.0 (79.3–100.0)	0.2373
X	26	86.7 (74.5–98.8)	2	6.7 (0.0–15.59)	<0.001
Y	30	100.0 (100.0–100.0)	27	90.0 (79.3–100.0)	0.2373
Seroprotection ≥ 8^b^					
Day 0					
A	16	53.3 (35.5–71.2)	18	60.0 (42.5–77.5)	0.7948
C	6	20.0 (5.7–34.3)	5	16.7 (3.3–30.0)	1.0000
W	6	20.0 (5.7–34.3)	6	20.0 (5.7–34.3)	1.0000
X	6	20.0 (5.7–34.3)	7	23.3 (8.2–38.5)	1.0000
Y	5	16.7 (3.3–30.0)	8	26.7 (10.8–42.5)	0.5321
Day 28					
A	30	100.0 (100.0–100.0)	28	93.3 (84.4–100.0)	0.4915
C	30	100.0 (100.0–100.0)	24	80.0 (65.7–94.3)	0.0237
W	30	100.0 (100.0–100.0)	27	90.0 (79.3–100.0)	0.2373
X	29	96.7 (90.2–100.0)	5	16.7 (3.3–30.0)	<0.001
Y	30	100.0 (100.0–100.0)	28	93.3 (84.4–100.0)	0.4915
Seroprotection ≥ 128^c^					
Day 0					
A	13	43.3 (25.6–61.1)	16	53.3 (35.5–71.2)	0.6058
C	3	10.0 (0.0–20.7)	2	6.7 (0.0–15.6)	1.0000
W	5	16.7 (3.3–30.0)	3	10.0 (0.0–20.7)	0.7065
X	4	13.3 (1.2–25.5)	3	10.0 (0.0–20.7)	1.0000
Y	4	13.3 (1.2–25.5)	8	26.7 (10.8–42.5)	0.3334
Day 28					
A	30	100.0 (100.0–100.0)	27	90.0 (79.3–100.0)	0.2373
C	30	100.0 (100.0–100.0)	24	80.0 (65.7–94.3)	0.0237
W	30	100.0 (100.0–100.0)	27	90.0 (79.3–100.0)	0.2373
X	29	96.7 (90.2–100.0)	5	16.7 (3.3–30.0)	<0.001
Y	30	100.0 (100.0–100.0)	27	90.0 (79.3–100.0)	0.2373

Data are presented as the percentage of participants (95% confidence interval). The confidence interval was estimated by the normal approximation method. *p*-values were calculated using the Chi-squared test or Fisher’s exact test.

*rSBA* baby rabbit complement serum bactericidal antibody assay, *CI* confidence interval.

^a^The proportion of participants who had an increase of at least 4 times rSBA titer at day 28.

^b^The proportion of participants with an rSBA titer of ≥8.

^c^The proportion of participants with an rSBA titer of ≥128.

After vaccination, 29 (96.7%) of 30 participants in the EuNmCV-5 group had titers of seroprotection (rSBA titer of ≥8, or ≥128) for serogroup X, and 30 (100.0%) for all other serogroups. In the MenACWY-CRM group, 24 (80.0%) to 28 (93.3%) of 30 participants had titers of seroprotection for serogroups A, C, W, and Y. As expected, few number of participants (5 (16.7%) of 30 participants) showed seroprotection for serogroup X. No significant differences were observed in seroprotection rates for serogroups A, W, and Y between the EuNmCV-5 and MenACWY-CRM groups. However, seroprotection rates were higher in the EuNmCV-5 group for serogroups C and X (Table 3).

**Fig. 2:**
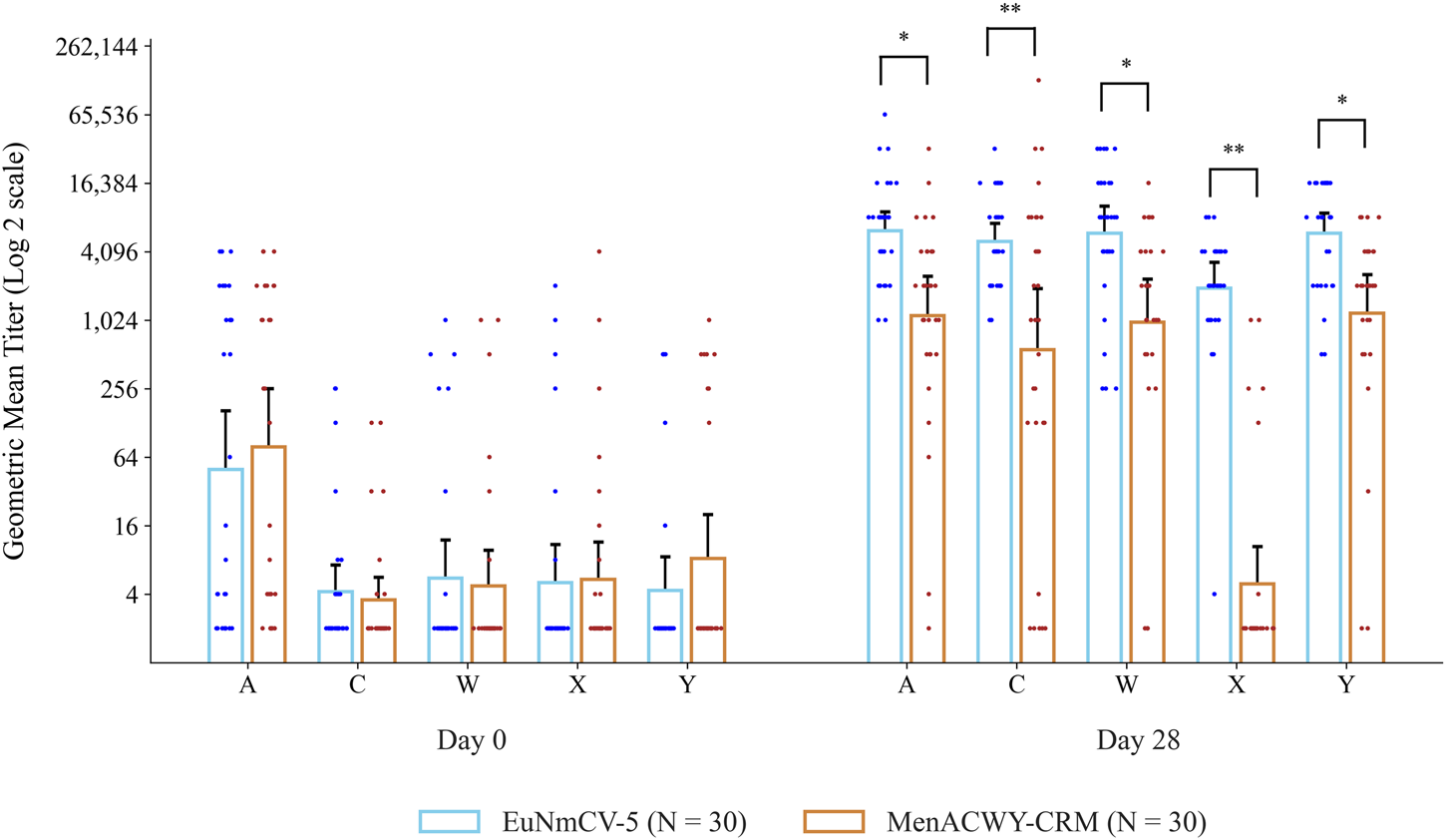
Geometric mean titers of each serogroup assessed by rSBA 28 The geometric mean titers and 95% confidence intervals (I bar) of the serum bactericidal antibody with rabbit complement (rSBA) are plotted on a logarithmic scale. 95% confidence intervals were computed using the normal approximation method. Statistical significance is indicated as follows: * = *p* < 0.001, ** = *p* < 0.0001.

At baseline, there were no significant differences in GMT against all serogroups between the EuNmCV-5 and MenACWY-CRM groups (Fig. 2). Notably, the EuNmCV-5 group demonstrated a significantly greater increase in GMT on day 28 than the MenACWY-CRM group for all serogroups. The GMRs (95% CIs) at day 28 were 5.5 (2.3–13.2), 9.0 (2.5–32.4), 6.1 (2.2–16.7), 397.1 (161.8–974.4), 5.0 (2.1–12.0) for serogroup A, C, W, X, and Y, respectively (all of the *p* < 0.001). Across all serogroups, the Geometric mean fold increase (GMFI) values were consistently higher for the EuNmCV-5 group. The GMFR values were found to be 8.8, 7.6, 5.2, 425.6, and 9.6, respectively (Table 4).

**Table 4:** Geometric mean fold increase of each serogroup assessed by rSBA 28 days after vaccination

Serogroup	EuNmCV-5	MenACWY-CRM	GMFR
	(*N* = 30)	(*N* = 30)	
A	125.1 (36.8–425.5)	14.3 (4.8–42.5)	8.8 (1.8–43.7)
C	1203.8 (669.0–2165.9)	157.6 (45.7–543.9)	7.6 (2.0–30.0)
W	1072.4 (506.3–2271.6)	207.9 (77.1–560.9)	5.2 (1.5–17.5)
X	388.0 (157.6–955.1)	0.9 (0.5–1.6)	425.6 (152.0–1192.0)
Y	1382.8 (639.6–2989.6)	143.7 (55.3–373.1)	9.6 (2.9–32.0)

The geometric mean fold increases (GMFIs) of each serogroup were presented along with their 95% confidence intervals, estimated using the normal approximation method. The geometric mean fold increases ratio (GMFR) represents the ratio between the GMFI in EuNmCV-5 and that in MenACWY-CRM for each respective serogroup.

*rSBA* baby rabbit complement serum bactericidal antibody assay, *GMFI* geometric mean fold increase, *CI* confidence interval, *GMFR* geometric mean fold increase ratio.

## Discussion

The study demonstrated that EuNmCV-5, the new pentavalent vaccine, was safe and well-tolerated in healthy Korean adults. No significant safety issues related to the vaccines were identified. The incidence and severity of both local and systemic reactions were found to be comparable between the two study vaccines. Both solicited and unsolicited adverse events were mild or moderate in severity and resolved without sequelae.

The SBA assay, using either rabbit (rSBA) or human (hSBA) sera, has demonstrated a high correlation with immunity to invasive meningococcal disease and is globally recommended as a regulatory standard for licensure of all meningococcal vaccines^11,12^. Immunogenicity assessment for meningococcal vaccines targeting serogroups A, C, W, or Y typically evaluates the proportion of participants who achieved more than fourfold rise in rSBA titers for each serogroup (seroconversion rate) or the proportion of participants who achieved rSBA titers above a predefined threshold (seroprotection rate). A conservative threshold for titers is considered as ≥1:8 in seronegative individuals and ≥1:128 in seropositive individuals^13–15^. In this study, we applied the same criteria to evaluate the immunogenicity of the study vaccines against serogroup X. This approach has been employed in previous studies on another meningococcal conjugate vaccine targeting serogroup X^13,16^. After 28 days following a single dose of vaccination, EuNmCV-5 induced seroconversion in more than 85% of participants for all the targeted serogroups, including serogroup X. This strong immune response was further demonstrated by the proportion of participants achieving a seroprotective titer. Moreover, the GMTs for all serogroups were significantly higher in the EuNmCV-5 group compared to the MenACWY-CRM group.

Some participants had high baseline titers before vaccination, suggesting that some participants might have had prior subclinical infections or acquired natural immunity. However, the baseline titers for each serogroup were comparable to or lower than those previously reported in other meningococcal vaccine studies in South Korea^17,18^. Two participants in the MenACWY-CRM group showed seroconversion against serogroup X. A possible explanation could be intraindividual variability in serogroup X antibodies or the presence of cross-reactive antibodies.

Taking all these factors into account, EuNmCV-5 appears to be an easily administered, safe, and potent vaccine candidate. Given the limited sample size and short-term follow-up period in this study, further investigations are needed to evaluate the long-term persistence of antibody levels and to monitor delayed adverse events. Additionally, the next phase 2/3 study of EuNMCV-5 will be conducted in Gambia and Mali. This study will include participants from a broader age range—healthy infants, toddlers, children, adolescents, and adults. It will evaluate the vaccine’s safety and efficacy across various age groups in areas where meningococcal disease is more prevalent.

## Methods

### Study design

A randomized, single-center, active-controlled, double-blind, first-in-human, phase 1 study was conducted to evaluate the safety, tolerability, and immunogenicity of EuNmCV-5, a pentavalent meningococcal conjugate vaccine in healthy Korean adults. The study was conducted in accordance with the Good Clinical Practice Guidelines and the principles outlined in the Declaration of Helsinki. The protocol was approved by the Institutional Review Board at Seoul National University Hospital (Seoul, Republic of Korea), and all participants provided written informed consent before enrolling in the study.

Eligible participants were randomly assigned in a 1:1 ratio to either the intervention vaccine, EuNmCV-5, or the control vaccine, MenACWY-CRM, and received a single injection of one of the two study vaccines. Due to visual differences between the two study vaccines, unblinded site clinical trial pharmacists and vaccinators were designated. An independent statistician generated a random allocation list in SAS^®^ software version 9.4 (SAS Institute, Cary, NC, USA), employing block randomization with predetermined block sizes (4 or 6). The random allocation list was sealed in envelopes and provided only to the unblinded clinical trial pharmacists. Unblinded clinical trial pharmacists filled all study vaccines into identical-appearing syringes according to the random allocation list in a secure location. Unblinded vaccinators then administered the allocated drugs to each participant. The unblinded clinical trial pharmacists and vaccinators did not participate in any other procedures.

After vaccination, participants were monitored for 30 min for anaphylactic reaction. Clinic visits were scheduled 7 and 28 days after vaccination. A follow-up telephone contact was made 180 days after vaccination (Fig. 1). Diary cards were distributed to participants on the vaccination day and 7 days after to record the duration and severity of solicited and unsolicited adverse events. Solicited adverse events were recorded for the first 7 days, and unsolicited adverse events were recorded through 28 days. The blinded site staff collected data from the diary cards during the scheduled clinic visits. Serious adverse events were tracked through 180 days after vaccination via telephone contact by the blinded site staff.

Blood samples for immunological assessment were collected before vaccination and 28 days after vaccination. The 28-day interval was chosen based on the previous studies on another meningococcal conjugate vaccine, which demonstrated that this timeframe allowed sufficient time for the formation of antibodies^13,19^.

### Study drugs

The intervention vaccine was EuNmCV-5, a pentavalent meningococcal CRM_197_-conjugated vaccine containing serogroups A, C, W, X, and Y. The control vaccine was MenACWY-CRM (Menveo^®^, GlaxoSmithKline, North Carolina, USA), a quadrivalent meningococcal CRM_197_-conjugated vaccine containing serogroups A, C, W, and Y. A quadrivalent vaccine was selected as control as there was no available pentavalent vaccine when the study was designed. EuNmCV-5 was contained in a single vial with a liquid component comprising 10 μg of meningococcal serogroup A and 5 μg each of serogroup C, W, X, and Y. The total recombinant CRM_197_ protein in EuNmCV-5 was ~49.5 μg/0.5 mL. MenACWY-CRM was composed of two vials. The first vial contained a powder with 10 μg of meningococcal serogroup A, while the second vial contained a liquid component of 5 μg each of serogroup C, W, and Y. The recombinant CRM_197_ protein in MenACWY-CRM was 25.4–65.5 μg/0.5 mL^20^. Just before the administration of MenACWY-CRM, the contents of the first vial were combined with the second vial. Both vaccines were administered as a single dose of 0.5 mL into the deltoid muscle.

### Study population

A total of 60 healthy Korean male and female participants, aged 19–55, were recruited for this study. A sample size of 60 was deemed appropriate for this first-in-human study to provide a descriptive evaluation of the safety, tolerability, and immunogenicity of EuNmCV-5. This was based on the participant numbers used for a previous phase 1 study on another meningococcal conjugate vaccine^13^.

Individuals with a history of meningococcal infection or in contact with a person with meningococcal infection within the last 2 weeks were excluded from the study based on self-reporting during the interview. Individuals who had received a previous meningococcal vaccination or had been vaccinated with other vaccines within the last 4 weeks were also excluded. Additionally, individuals with any chronic medical conditions, immunodeficiency, or a history of hypersensitivity to any vaccination were excluded from participation. Through self-reporting during the interview, those who experienced fever (≥38 °C) within 3 days of screening or had a significant acute or chronic infection within 7 days were excluded. Individuals with positive urine drug screens, positive blood screens for hepatitis B/C or HIV, clinically significant laboratory abnormalities (including liver function tests), and positive pregnancy tests were also excluded from the study.

### Safety assessment

Safety and tolerability were assessed by clinical laboratory tests, physical examination, vital signs, and monitoring of solicited and unsolicited adverse events. During the initial 7 days following vaccination, participants reported solicited adverse events. Solicited adverse events included both systemic reactions (fever, headache, fatigue/malaise, nausea, vomiting, myalgia, arthralgia, chill, rash, and acute allergic reaction) and local injection-site reactions (pain, tenderness, erythema/redness, induration/swelling, and urticaria). Erythema/redness or induration/swelling was classified as life-threatening if necrosis occurred, severe if the diameter was ≥10.0 cm, moderate if 5.1–9.9 cm, and mild if ≤5.0 cm. Fever was classified as life-threatening if the temperature was >40.0 °C, severe if 39.0–40.0 °C, moderate if 38.5–38.9 °C, and mild if 38.0–38.4 °C. Other adverse events were graded as follows: mild (no interference with normal activities), moderate (some interference with normal activities), severe (prevention of normal activities), and life-threatening.

Safety parameters included anaphylactic reaction occurring within 30 min post-vaccination, solicited adverse events recorded within 7 days post-vaccination, unsolicited adverse events documented within 28 days post-vaccination, and serious adverse events monitored over 180 days following vaccination.

### Immunogenicity assessment

For the immunogenicity assessment, ~20 mL of blood was collected before vaccination (day 0) and day 28, using serum-separating tubes and stored at room temperature for a minimum of 30 minutes to a maximum of 2 hours. Blood samples were centrifuged at 4 °C and 1900 *g* for 20 minutes. Approximately 1.0 mL of supernatant was stored at −70 °C until analysis. The serum samples were tested with a rabbit complement-dependent serum bactericidal antibody (rSBA) assay. The UK Health Security Agency Vaccine Evaluation Unit (Manchester, United Kingdom) measured the rSBA titers against five target serogroups: A, C, W, X, and Y. The target strains in the rSBA assays were serogroup A F8238, serogroup C C11, serogroup Y s1975, serogroup W M01 240070, and serogroup X BF 2/97. The rSBA assay method was adapted as previously published^21^. The lower limit of quantitation (LLOQ) was set at a titer of four. Antibody titers below LLOQ were considered to have a value of half the LLOQ.

Immunogenicity parameters included the proportion of participants with seroconversion, defined as individuals who had an increase of at least 4 times rSBA titer at day 28. This was characterized as a post-vaccination titer of ≥32 for seronegative participants whose baseline titer is <8 or a ≥ 4-fold increase in post-vaccination titer for seropositive participants whose baseline titer is ≥8. Additionally, the proportion of participants achieving seroprotection was defined as individuals with an rSBA titer of ≥8 at day 28 or those with an rSBA titer of ≥128 at day 28. The seroprotection threshold has been validated as an effective indicator of protection in both seronegative and seropositive individuals in prior studies of other meningococcal conjugate vaccines^13–15^. Furthermore, geometric mean titers (GMTs) for each serogroup at day 28 were assessed, along with GMT ratios (GMRs) comparing the EuNmCV-5 and MenACWY-CRM groups. GMFI from baseline and their ratios (geometric mean fold increase ratio, GMFR) between the two groups were also presented for each serogroup.

### Statistical analysis

Safety data were analyzed for the participants who were administered the study vaccines. Solicited and unsolicited adverse events were analyzed by the number and percentage of participants experiencing each event.

Immunogenicity data were analyzed for the participants who received the study vaccines and with post-vaccination immunogenicity data. The immunogenicity parameters of each serogroup were summarized separately based on the treatment. GMTs were calculated by transforming the titers to a logarithmic scale, computing the mean on the transformed scale, and converting the mean value back to the original scale. To assess the differences in GMTs between the two study vaccines, 95% confidence intervals (CIs) were computed using the normal approximation method. Additionally, independent t-tests were conducted on the log2-transformed titers to compare the means. The seroconversion rate and seroprotection of the two study vaccines, along with their 95% CIs, were calculated using the normal approximation method. Seroconversion rate and seroprotection rates were compared between treatment groups using the Chi-squared test or Fisher’s exact test. Significance was set at *p* < 0.05 (two-sided). All data analyses and statistical computations were done with SAS^®^ software version 9.4 (SAS Institute, Cary, NC, USA).

## Data Availability

Due to the privacy of study participants, the data supporting this study’s findings are not openly available. However, they are available from the corresponding author upon reasonable request.
