# Author Correction: A randomized study to evaluate the safety and immunogenicity of a pentavalent meningococcal vaccine

**DOI:** 10.1038/s41541-024-00965-2

**Published:** 2024-09-19

**Authors:** Yoonjin Kim, Sungyeun Bae, Kyung-Sang Yu, SeungHwan Lee, Chankyu Lee, Jinil Kim, Howard Her, Jaeseong Oh

**Affiliations:** 1 Department of Clinical Pharmacology and Therapeutics, Seoul National University College of Medicine and Hospital, Seoul, Republic of Korea; 2 R&D Division, EuBiologics Co., Ltd, Seoul, Republic of Korea; 3 Department of Pharmacology, Jeju National University College of Medicine, Jeju, Republic of Korea; 4 Clinical Research Institute, Jeju National University Hospital, Jeju, Republic of Korea

**Keywords:** Drug development, Meningitis

Correction to: *npj Vaccines* 10.1038/s41541-024-00935-8, published online 07 August 2024

The original version of this article contained an error in the name of the analytical laboratory for immunogenicity assessment. The correct name is the UK Health Security Agency Vaccine Evaluation Unit (Manchester, United Kingdom), but it was incorrectly stated as the Department of Health and Social Care at the UK Health Security Agency (UKHSA, London, UK). This error has now been corrected in both the PDF and HTML versions of the article.

